# An improved calibration and uncertainty analysis approach using a multicriteria sequential algorithm for hydrological modeling

**DOI:** 10.1038/s41598-021-96250-6

**Published:** 2021-08-20

**Authors:** Hongjing Wu, Bing Chen, Xudong Ye, Huaicheng Guo, Xianyong Meng, Baiyu Zhang

**Affiliations:** 1 Northern Region Persistent Organic Pollution Control (NRPOP) Laboratory, Faculty of Engineering and Applied Science, Memorial University of Newfoundland, St. John’s, NL A1B 3X5 Canada; 2 College of Environmental Science and Engineering, Peking University, No. 5 Yiheyuan Road Haidian District, Beijing, 100871 People’s Republic of China; 3 School of Applied Meteorology, Nanjing University of Information Science and Technology, Nanjing, 210044 People’s Republic of China

**Keywords:** Hydrology, Environmental impact

## Abstract

Hydrological models are widely used as simplified, conceptual, mathematical representatives for water resource management. The performance of hydrological modeling is usually challenged by model calibration and uncertainty analysis during modeling exercises. In this study, a multicriteria sequential calibration and uncertainty analysis (MS-CUA) method was proposed to improve the efficiency and performance of hydrological modeling with high reliability. To evaluate the performance and feasibility of the proposed method, two case studies were conducted in comparison with two other methods, sequential uncertainty fitting algorithm (SUFI-2) and generalized likelihood uncertainty estimation (GLUE). The results indicated that the MS-CUA method could quickly locate the highest posterior density regions to improve computational efficiency. The developed method also provided better-calibrated results (e.g., the higher NSE value of 0.91, 0.97, and 0.74) and more balanced uncertainty analysis results (e.g., the largest P/R ratio values of 1.23, 2.15, and 1.00) comparing with other traditional methods for both case studies.

## Introduction

Hydrological models have been significantly improved over the past several decades and applied to different applications and water resource management, such as land use management^1^, flood prediction^2–4^, sediment transport simulation^5^, and nutrient yield simulation^6,7^. The complexity of hydrological models has been dramatically increased (e.g., from lumped models to distributed models), especially for distributed models^8^. The extensive data and computational requirement, along with large numbers of unadjusted distributed or semi-distributed parameters, always become the obstacles for improving hydrological modeling^9^. Various approaches have been developed to assess and enhance hydrological modeling performance. They included parameter optimization^10,11^, calibration^12,13^, design space exploration^14^, sensitivity analysis^15,16^, and uncertainty analysis^17–19^.

Most conceptual distributed and semi-distributed parameters cannot be easily determined due to the limitations of parameter measurements from physical systems and available data. Moreover, measurement errors and system uncertainties are inevitable. Therefore, model calibration should be applied to adjust parameters within recommended ranges by optimizing observed and simulated data^20^. Calibration is always challenging due to the uncertainties from model input, model structure, parameter, and output^21–23^. Uncertainty is a subjective factor of people’s confidence in the understanding of individuals and communities. Based on personal experiences, assumptions, and knowledge, different people will come to different conclusions on how uncertain something is^24^. Imperfect knowledge, especially in hydrological studies, makes uncertainty inevitable. As many distributed hydrological models are extensively used to support the decision-making process of watershed management, it is essential and necessary to perform careful calibration and uncertainties analysis for these modeling studies.

For hydrological studies, parameter uncertainty has always been given the most concerns. According to empirical estimations, the uncertainty of model parameters may compromise the reliability and precision of modeling results, such as physical significance, generalized inference, and observed data^25,26^. Moreover, the interactions between the parameters also cause uncertainties. For example, a single-valued parameter set results in a single output for modeling. However, in inverse applications, multiple combinations of different parameter sets can reproduce a same observed output value. This non-unique phenomenon is known as "equifinality", which is an inherent property of inverse modeling demonstrating the uncertainties of parameters^27^. For uncertainty reduction purposes, many existed obvious errors of parameters that can generate reasonable outputs need to be removed. Due to a large number of unknown or unmeasurable parameters and errors in the data, parameter uncertainty must be controlled and quantified^28^.

In the past several decades, several methods have been proposed to address parameter uncertainty. The most traditional statistical way is the first-order approximation, but one of the weaknesses is that the correlation between parameters cannot be evaluated^29^. Other methods for estimating the parameter confidence levels include the uniform grid sampling, uniform random sampling, and sequential uncertainty fitting algorithm (SUFI-2)^30–32^. These methods typically require massive computational resources for high-dimensional models. The sampling scheme may fail to search the global optimum among the parameter space if the initial range is not dense enough^29,33^. Monte Carlo based approaches, such as Markov Chain Monte Carlo (MCMC) simulation^34^ and generalized likelihood uncertainty estimation (GLUE)^35^, are widely used in hydrological modeling for quantifying parameter uncertainties. These methods can consider the nonlinearity and interdependency of parameters. However, the computational requirement is relatively high when facing complex nonlinear hydrological models (e.g., some complex distributed models)^36^.

The motivation of this study is to propose a method to improve efficiency and accuracy of calibration and uncertainty analysis for supporting hydrological modeling. Therefore, the multicriteria sequential calibration and uncertainty analysis (MS-CUA) method was proposed. The feasibility and performance of MS-CUA were evaluated using a hypothetical case provided by the SWAT calibration and uncertainty program (SWAT-CUP) and a real case study at the upstream of the Wenjing River watershed in Chongzhou, China. The calibration and uncertainty analysis results were compared with the results of GLUE and SUFI-2. The results indicated that the proposed MS-CUA method can achieve higher efficiency for parameter calibration and more balanced uncertainty analysis results than the other two methods.

## Results and discussion

Two case studies were conducted to evaluate the performance and efficiency of the proposed methods. The MS-CUA method was applied to simulation results for both cases to test the feasibility and flexibility. A hypothetical case was conducted with demo data for two outlets in a watershed from the SWAT Calibration and Uncertainty Programs (SWAT-CUP) (*Case 1*). The MS-CUA method was simplified by using only two iterations without adding any additional criterion, and the results were compared with GLUE. MS-CUA was also applied to a comprehensive real-world case of the upstream of the Wenjing River watershed in Chongzhou, China (*Case 2*). The real case study was conducted for four iterations, and the results are compared with the results from SUFI-2 and GLUE. The results performance of applying MS-CUA from the two cases were comprehensively evaluated and analyzed from a simplified and conceptual point to a complicated and practical aspect.

### Case 1: A hypothetical case using the demo data from SWAT-CUP

The simplified MS-CUA method was applied to a hypothetical case as a preliminary test using the demo data from SWAT-CUP. The Nash-Sutcliffe efficiency (NSE) was used as the likelihood function. The performance improvements of calibration were tested using two iterations with 1000 simulations in each iteration. The threshold value was set to 0.8 due to the high match between the simulated and observed surface runoff. In the study, over 35% of observation should be included in 95 percent prediction uncertainty (95PPU) iteratively to achieve acceptable uncertainty analysis results. Ten considered parameters included CN_2_, ALPHA_BF, GW_DELAY, CH_N2, CH_K2, ALPHA_BNK, SOL_AWC, SOL_K, SOL_BD, and SFTMP. The definition of each parameter has been provided in Table 1.

**Table 1:** Definitions of selected input parameters in SWAT ^37^.

Parameters	Definition
CN_2_	Initial SCS runoff curve number for moisture condition II
ALPHA_BF	Baseflow alpha factor (days)
GW_DELAY:	The delay time, δ_gw_, cannot be directly measured
GWQMN:	Threshold depth of water in the shallow aquifer required for return flow to occur (mm H20)
ESCO	Soil evaporation compensation factor
CH_N2	Manning's "n" value for the main channel
CH_K2:	Effective hydraulic conductivity in main channel alluvium (mm/hr)
ALPHA_BNK	Baseflow alpha factor for bank storage (days)
SOL_AWC:	Available water capacity of the soil layer (mm H2O/mm soil)
SOL_K	Saturated hydraulic conductivity for the first soil layer
SOL_BD	Moist bulk density for the first soil layer
SFTMP:	Snowfall temperature (°C)
GW_REVAP	Groundwater "revap" coefficient
RCHRG_DP:	Deep aquifer percolation fraction

**Figure 1:**
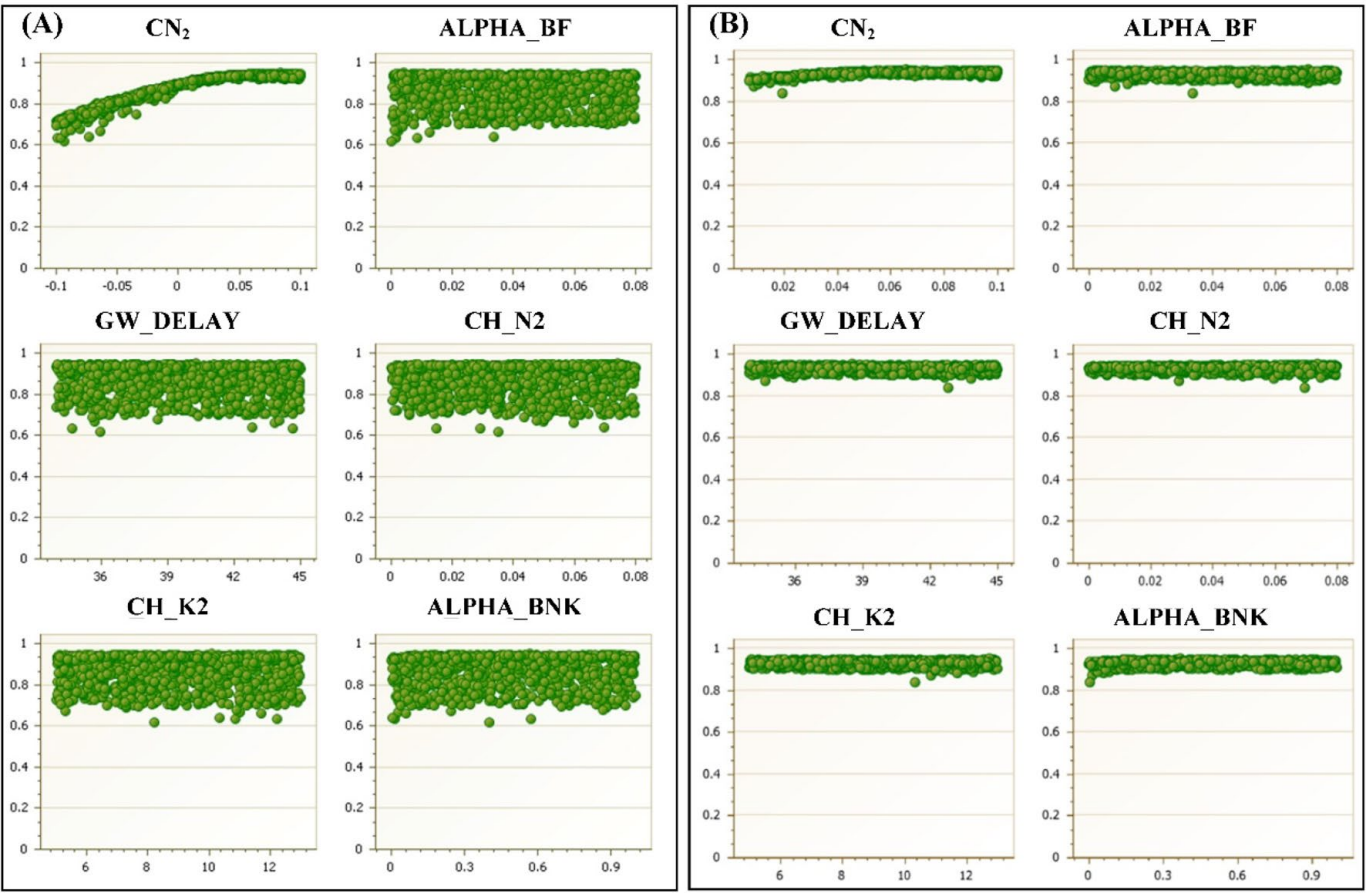
Parameter ranges of selected parameters in the first iteration (**A**) and second iteration (**B**).

In this study, to make analysis results comparable and keep consistency, the Latin Hypercube Sampling (LHS) method was adopted as the sampling scheme to show the feasibility of the proposed method. Therefore, the LHS method was applied to generate 2000 parameter sets (for two iterations) from prior uniform distributions of each parameter. After the first iteration, the parameter ranges were adjusted according to the NSE results for multi-iteration performance evaluation. The examples of NSE values verse some parameters for iterations 1 and 2 were shown in Fig. 1. The 95PPU of surface runoff was obtained through the first iteration, and the new parameter ranges were updated based on ranking the behavioural parameter sets correspondingly. For example, from the plot for parameter CN_2_ in Fig. 1A, it is shown that there is an apparent curvature within the original predefined parameter space, and the NSE value increases with the increase of CN_2_ value. Therefore, the desirable solutions should locate in the region of larger values of CN_2_ within the predefined feasible space. Through evaluating the likelihood value of the objective function, some impractical parameter sets were removed from behavioural parameter sets. By using refined behavioural parameter sets, the 95PPU and parameter ranges were updated. As a simplified case, only parameter CN_2_ was adjusted for the second iteration. As shown in Fig. 1B, the lower bound of parameter CN_2_ was shifted from − 0.10 to − 0.01.

The comparison results between the simplified MS-CUA in 2000 simulation scenarios and GLUE in 10,000 simulation scenarios were shown in Table 2. In Outlet 1, the best simulation of the MS-CUA method and the GLUE method reached 0.91 and 0.90 for the value of NSE, respectively. Both methods achieved the same R^2^ values of 0.91. The results represented that the calibrated simulations from both methods were very close to the observed surface runoff due to the high NSE and R^2^ values. In this study, besides the goodness of calibration, the performance of uncertainty analysis is quantified and evaluated using the P/R ratio for different methods. Using the P/R ratio is an efficient way to show the strength and performance of uncertainty analysis. P-factor is defined as the percentage of observations covered by the 95PPU and R-factor is used to evaluate the relative width of 95PPU band ^24^. The larger P-factor value means more observed data were bracketed by the 95PPU indicating better prediction results. However, if the 95PPU band is too large, the 95PPU can cover almost all observed values. Because of the large 95PPU band, the prediction uncertainty can become extremely large and meaningless for decision-making and analysis. The good uncertainty analysis searched for the results that bracketed most of the observed data with the smallest possible uncertainty band, which means the good uncertainty analysis results should have a relatively large P-factor value with a relatively small R-factor value. Therefore, the greater the ratio of P/R is, the better the simulation performance achieved. The MS-CUA method provided a smaller R-factor value of 0.30 than the GLUE method (R-factor = 0.53) with the same P-factor value of 0.38. The P/R ratio using MS-CUA in Outlet 1 is 1.23, which is much larger than the ratio value of 0.72 by using GLUE. Although the results for Outlet 1 barely showed the improvement of MS-CUA over GLUE for the best-calibrated simulation, the ratio of P-factor and R-factor indicated that the MS-CUA method provided better uncertainty analysis results than GLUE. In Outlet 2, the best surface runoff simulation results from the second iteration also indicated that the proposed MS-CUA achieved better results than those from GLUE with 10,000 simulation runs. The NSE of MS-CUA and GLUE were 0.97 and 0.96, R^2^ values of the two methods were 0.98 and 0.97, respectively. They indicated that MS-CUA had a better calibration performance. The P/R ratio of MS-CUA was 2.15, which was much greater than the value from GLUE (1.34). That represented that MS-CUA had a better uncertainty analysis result.

**Table 2:** The comparison results between the simplified MS-CUA method and GLUE.

Outlet	Variable	Behavioural simulation	P-factor	R-factor	R^2^	NSE	P/R
1	1st iteration of MS-CUA	484 (1,000)	0.38	0.31	0.89	0.88	1.23
	2nd iteration of MS-CUA	976 (1,000)	0.38	0.3	0.91	0.91	1.23
	GLUE	7660 (10,000)	0.38	0.53	0.91	0.90	0.72
2	1st iteration of MS-CUA	484 (1,000)	0.58	0.27	0.97	0.96	2.15
	2nd iteration of MS-CUA	976 (1,000)	0.58	0.27	0.98	0.97	2.15
	GLUE	7660 (10,000)	0.63	0.47	0.97	0.96	1.34

*P-factor: the percentage of observations covered by the 95PPU.

*R-factor: the measure of the relative width of 95% probability band.

Table 2 also showed the results of the first iteration with original parameter ranges and the second iteration with the updated parameter range (only using updated parameter ranges of CN_2_ as an example) with the MS-CUA method. The second iteration improved the NSE and R^2^ of the best simulation for Outlet 1 and Outlet 2. Due to the high quality of simulation results, limited improvement was found for the best simulation results in the second iteration. However, through the simplified MS-CUA method, the number of behavioural simulations dramatically increased with the updated parameter ranges. The percentages of behavioural parameter sets among the total parameter sets were larger than the corresponding percentages in GLUE. It indicated that the MS-CUA method could more accurately capture the highest posterior density (HPD) region than the random Monte Carlo sampling method of GLUE. The reasonable and acceptable results can be achieved at the circumstance of dramatically reducing parameter uncertainties indicating the advantages of the proposed MS-CUA method.

The above analysis clearly showed that the MS-CUA method could provide better calibration and uncertainty analysis results than the traditional GLUE method. At the same time, MS-CUA was far more efficient compared with GLUE. Only 2000 simulation runs were conducted using MS-CUA and achieved better calibration and uncertainty analysis results in comparison with the 10,000 simulation runs by GLUE. The computational time is directly correlated with the number of simulation runs. Therefore, in this study, MS-CUA only took 20% of the time used by GLUE, indicating the advantages of the proposed method.

Another significant advantage of MS-CUA is that the parameter uncertainty can be reduced by using updated parameter ranges. Therefore, the MS-CUA method can help to reduce uncertainty effectively. The uncertainty reduction is very meaningful, especially for uncertainty propagation problems.

### Case 2: A real case study at the upstream of Wenjing River watershed

**Figure 2:**
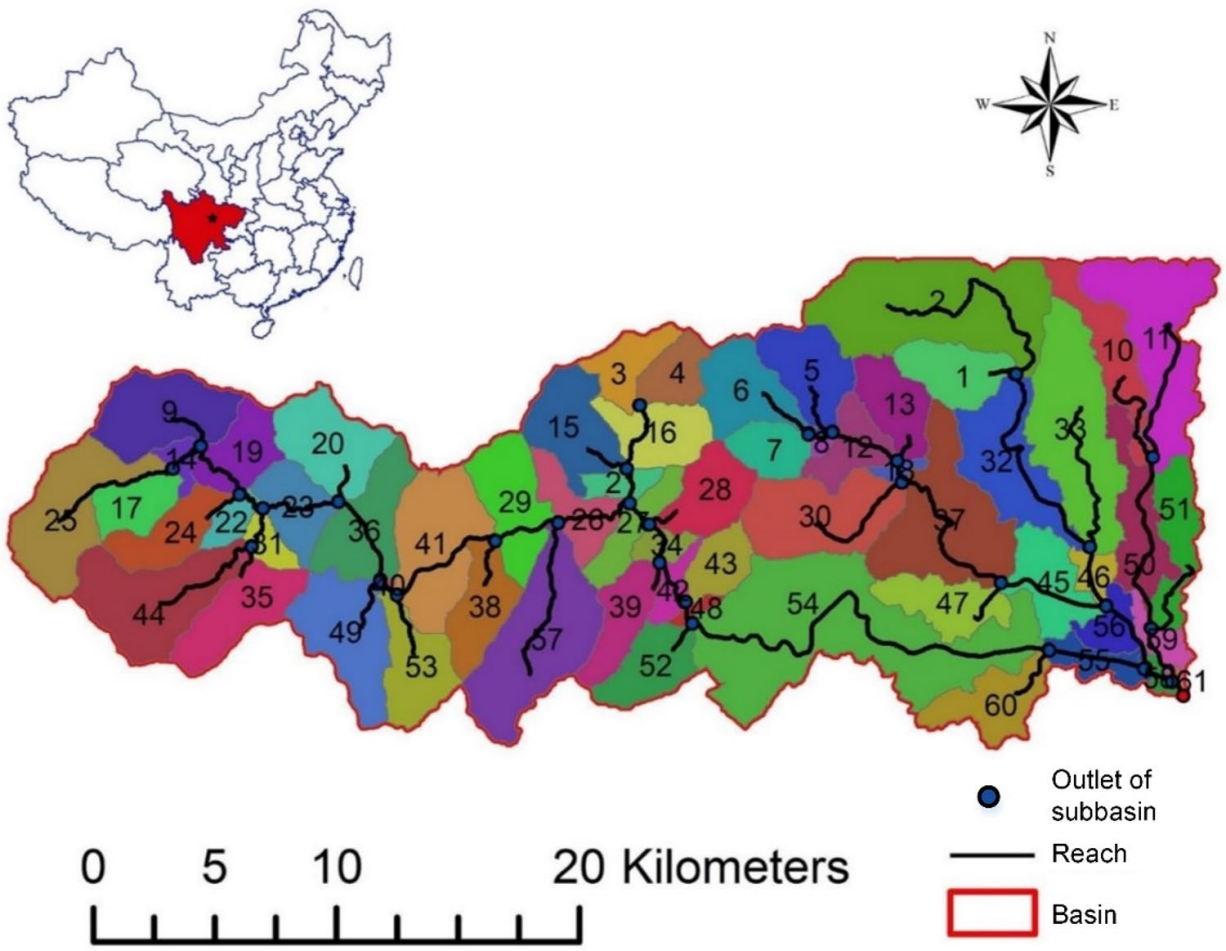
Location map of the upstream of the Wenjing River watershed 12. The map is generated with ArcMap Version 10.5.

A real case study was conducted to evaluate the feasibility and performance of the proposed MS-CUA method. The upstream of the Wenjing River watershed was selected as the study area. Figure 2 showed the watershed location and digital elevation model (DEM) of the study area^12^.

- Results of MS-CUA

The MS-CUA method conducted four iterations with 1000 simulation runs per iteration. To get reasonable uncertainty analysis, an assumption has been made that at least 35% of observed surface runoff should be included in 95PPU for each iteration. A total of 11 parameters involved in calibration and uncertainty analysis, as well as definitions of each parameter, were provided in Table 1. Two comparable scenarios of simulations with one or two criteria were conducted to investigate the performance.

### Simulations using one criterion

The statistical summary of simulation results was shown in Table 3. In the simulation results with one criterion, the threshold values of the objective function for four iterations were set as different values (0.20, 0.40, 0.60 and 0.60). Since it was difficult to determine the HPD region for the first iteration, a relatively low threshold of the objective function was set for the preliminary HPD estimation of different parameter ranges. When approaching the HPD to obtain smaller parameter ranges, the objective function threshold can be adjusted for each iteration. A higher threshold would result in a smaller number of behavioural parameters. Thus, the threshold value was set to 0.60 for the fourth iteration to guarantee reasonable parameter ranges for different parameters.

**Table 3:** The statistic summary of the simulation results.

Scenario	Iteration	No. of criteria	Threshold value	Behavioural parameter sets	P-factor	R-factor	R^2^	NSE	P/R
With one criterion	First	–	0.20	86	0.50	0.90	0.78	0.68	0.56
	Second	–	0.40	61	0.50	0.78	0.78	0.70	0.64
	Third	–	0.60	49	0.47	0.54	0.77	0.70	0.87
	Fourth	–	0.60	410	0.42	0.49	0.77	0.71	0.86
With two criteria	First	One	NSE = 0.20	86	0.50	0.90	0.78	0.68	0.56
		Two	NSE = 0.20, R^2^ = 0.60	81	0.50	0.90	0.78	0.68	0.56
	Second	One	NSE = 0.40	62	0.47	0.71	0.78	0.70	0.66
		Two	NSE = 0.40, R^2^ = 0.70	34	0.47	0.66	0.78	0.70	0.71
	Third	One	NSE = 0.60	74	0.44	0.52	0.78	0.73	0.85
		Two	NSE = 0.60, R^2^ = 0.75	41	0.42	0.45	0.78	0.73	0.93
	Fourth	One	NSE = 0.60	605	0.39	0.39	0.79	0.74	1.00
		Two	NSE = 0.60 R^2^ = 0.75	527	0.39	0.39	0.79	0.74	1.00

After each iteration, the parameter sets generating 95PPU of surface runoff were used to update parameter ranges for the next iteration. The parameter range values of each iteration were shown in Table 4. From the first to fourth iteration, the number of behavioural parameter sets increased from 86 to 410, indicating the updated parameter ranges are around the HPD region after four iterations. To further investigate the performance of calibration and uncertainty analysis using the MS-CUA method, the P-factor, R-factor, and R^2^ and NSE values of the best simulation in each iteration were shown in Table 3. Typically, the smaller uncertainty bands resulted in a smaller coverage of observed runoff data. Therefore, the larger value of P/R with acceptable coverage was the desired result. Although the P-factor slightly decreased from 0.5 to 0.42 during iterations, the R-factor was dramatically decreased from 0.90 to 0.49 simultaneously. The P/R ratio increased from 0.56 to 0.86, which demonstrated the improvement of uncertainty analysis results. The R^2^ values of four iterations are pretty constant with a high value (above 0.77), showing a good correlation between simulated results and observed surface runoff. The NSE values for four iterations increased from 0.68 to 0.71, and a better-calibrated simulation was achieved. Through the application of the MS-CUA method with one criterion, the ratio of P/R, R^2^ and NSE all increased during the multiple iterations, indicating the improvement of calibration and uncertainty analysis using MS-CUA.

**Table 4:** Parameter range values of each iteration.

Parameter	Scenario*	First iteration		Second iteration		Third iteration		Fourth iteration	
		LB**	UB	LB	UB	LB	UB	LB	UB
CN2	1	− 0.25	0.30	− 0.22	0.29	− 0.19	0.28	− 0.19	0.13
	2	− 0.25	0.30	− 0.22	0.29	− 0.18	0.25	− 0.18	0.07
ALPHA_BF	1	0.40	1.00	0.43	0.99	0.44	0.97	0.48	0.92
	2	0.40	1.00	0.43	0.99	0.47	0.96	0.52	0.95
GW_DELAY	1	10.00	300.00	13.84	280.90	25.73	248.05	33.21	155.65
	2	10.00	300.00	13.63	281.30	22.83	169.09	38.56	102.33
GWQMN	1	0.00	2000.00	13.75	1358.25	21.82	586.51	31.45	429.00
	2	0.00	2000.00	13.00	1375.00	54.03	505.09	81.32	446.23
ESCO	1	0.80	1.00	0.81	0.99	0.83	0.99	0.84	0.99
	2	0.80	1.00	0.81	0.99	0.82	0.99	0.82	0.98
CH_K	1	5.00	130.00	10.81	128.17	16.45	121.83	19.10	118.34
	2	5.00	130.00	10.31	128.31	14.89	120.62	16.84	117.72
ALPHA_BNK	1	0.00	1.00	0.04	0.98	0.062	0.95	0.14	0.92
	2	0.00	1.00	0.04	0.98	0.088	0.88	0.12	0.83
SOL_AWC	1	− 0.20	0.40	− 0.18	0.39	− 0.13	0.38	− 0.12	0.36
	2	− 0.20	0.40	− 0.17	0.39	− 0.15	0.34	− 0.12	0.32
SFTMP	1	− 5.00	5.00	− 4.46	4.70	− 4.07	4.38	− 4.01	3.99
	2	− 5.00	5.00	− 4.25	4.71	− 4.02	4.59	− 3.89	4.01
GW_REVAP	1	0.02	0.50	0.02	0.44	0.026	0.24	0.03	0.15
	2	0.02	0.50	0.02	0.44	0.037	0.26	0.04	0.12
RCHRG_DP	1	0.00	1.00	0.0080	0.64	0.014	0.37	0.0145	0.19
	2	0.00	1.00	0.0075	0.64	0.019	0.34	0.0348	0.19

*Scenarios: 1, simulations with one criterion; 2, simulations with two criteria.

**LB: Lower bound; UB: Upper bound.

### Simulations using two criteria

To further test the proposed MS-CUA method, the simulation with two criteria was proposed to screen the behavioural parameter sets instead of one criterion. The R^2^ was used as an additional criterion to further screen parameter sets. These screened parameter sets are called "refined behavioural parameter sets" among the behavioural parameter sets. During each iteration, the results for behavioural parameter sets (screened by the objective function) and refined behavioural parameter sets (screened by the objective function and R^2^) in each iteration were also shown in Table 3. The threshold values of the objective function for four iterations were set as the same for the one criterion case (0.2, 0.4, 0.6, and 0.6). The R^2^ was used as the additional criterion in each iteration. Due to the good correlation between simulated runoff results and observed surface runoff, the values were set as 0.60, 0.70, 0.75, and 0.75 for the four iterations.

For the first iteration, the threshold value of the objective function was set to 0.20. Two situations were analyzed separately, including the results for behavioural parameters and the refined behavioural parameter sets in each iteration. The threshold value of the second criterion R^2^ was set to 0.60 for screening the refined behavioural parameters among the behavioural parameter sets. There are 81 refined behavioural parameter sets among 86 behavioural parameter sets in this iteration. Because only five parameter sets were removed from behavioural parameter sets due to the lower R^2^ values, P-factor and R-factor are the same for the two situations, which are 0.50 and 0.90, respectively. The values of R^2^ and NSE of the best-calibrated simulation are 0.79 and 0.68, indicating reasonably good simulation results. Because the best-calibrated simulation was not removed when using additional criteria, the R^2^ and NSE of the best simulation will not change for two situations within an iteration. According to the 95PPU of the 81 parameter sets (screened by using two criteria), the updated parameter ranges for the second iteration were calculated.

For the second iteration, the threshold value of the objective function increased to 0.40. The 62 behavioural parameter sets can be found. The R^2^ and NSE values of the best-calibrated simulation increased to 0.78 and 0.70, respectively. When the R^2^ was set to 0.70 for the second iteration, only 34 behavioural parameter sets were left as refined behavioural parameter sets. Therefore, the P-factor and R-factor were different for the two situations. The R-factor of two criteria (0.66) was smaller than that of one criterion (0.71), showing reducing the prediction uncertainty. The same P-factor value of 0.47 indicated that the reducing uncertainty did not affect the model prediction power and still covered the same number of observed data. The results with reduced uncertainty through two criteria were preferred results, and the 95PPU of the 34 refined behavioural parameter sets were used to calculate the parameter ranges for the third iteration.

For the third iteration, the threshold value of NSE was set to 0.60. There are 74 behavioural parameter sets after screening. The R^2^ and NSE values of the best-calibrated simulation were 0.78 and 0.73, respectively. The R^2^ and NSE values were greater than the results in the second iteration, indicating the improvement of the calibrated results. The additional criterion R^2^ was increased to 0.75 in this iteration for getting better refined behavioural parameter sets. Within 74 behavioural parameter sets, 41 parameter sets can achieve the value of R^2^ greater than 0.75. The R-factor reduced from 0.52 to 0.45 when using two criteria. Even though the P-factor reduced a little (from 0.44 to 0.42), the P-factor ratio and R-factor ratio increased from 0.85 to 0.93, showing the improvement of uncertainty analysis results. The 95PPU values of the 41 refined behavioural parameter sets were used to calculate the parameter ranges for the fourth iteration. The exact values of each parameter range for four iterations were shown in Table 4.

For the last iteration, the threshold values of the objective function and the additional criterion were set at the same value as the third iteration. Due to the application of the previous three iterations, the HPD region was estimated for each parameter. Therefore, the number of behavioural parameter sets was increased to 605, and the number of refined behavioural parameter sets was determined to 527. The R^2^ and NSE of the best-calibrated simulation are 0.79 and 0.74, respectively, which are the best-calibrated simulation among the four iterations. The R-factor decreased to 0.39, with a P-factor of 0.39 for both situations. The ratios of P-factor and R-factor are 1.00, which were the largest value of four iterations. All those values of different indicators showed that the last iteration achieved the best-calibrated simulation with the most balanced uncertainty analysis results.

**Figure 3:**
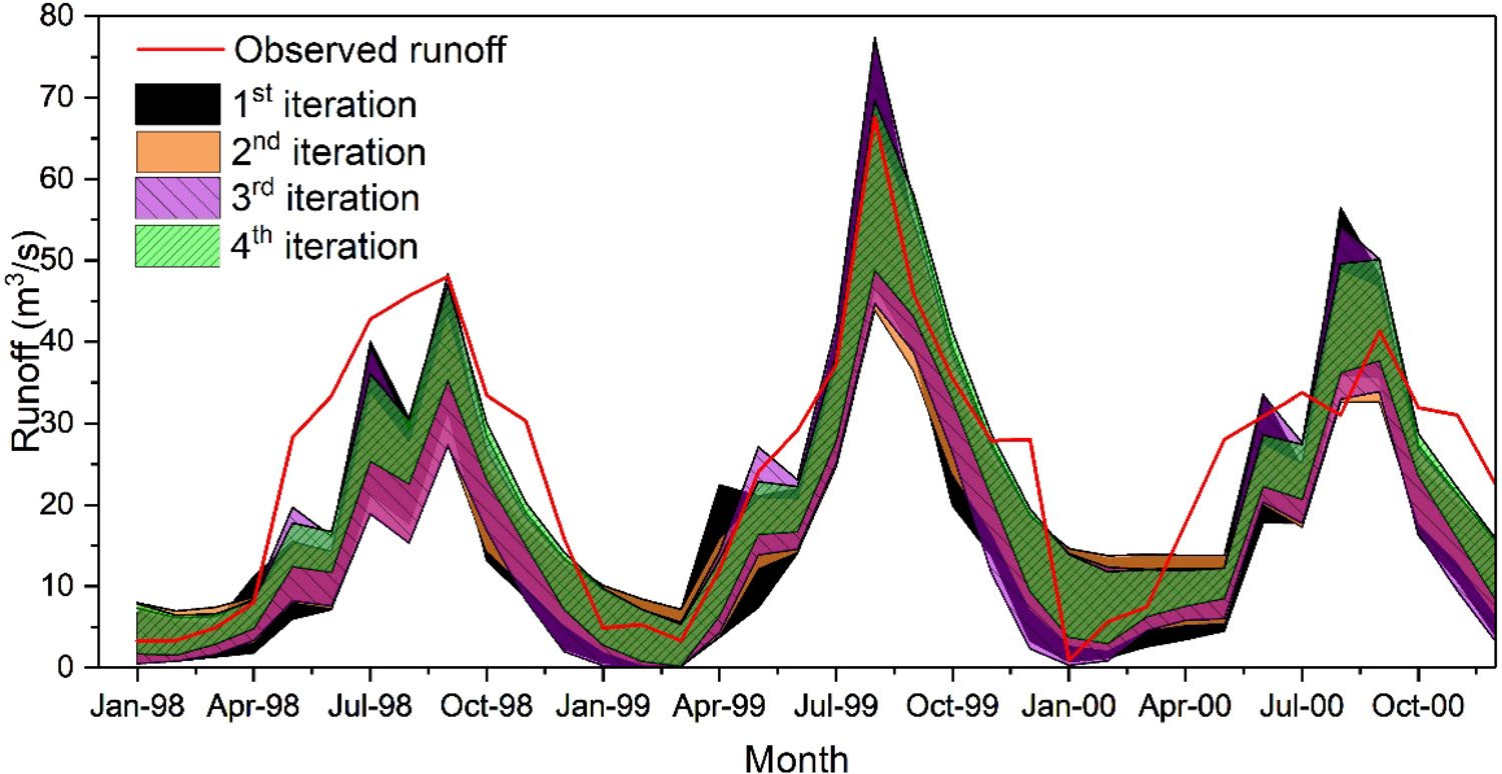
The 95PPU of surface runoff for four iterations using the MS-CUA method.

The MS-CUA method can effectively calibrate the hydrological model and provide reasonably good uncertainty analysis results through four iterations. The best-calibrated simulation results were improved in each iteration, and more balanced and reliable uncertainty analysis results were provided (larger ratio of P-factor and R-factor). The prediction uncertainty decreased in each iteration represented by the 95PPU of surface runoff. Through the multicriteria screen process, the behavioural parameter sets in each iteration were reduced, indicating the reduction of the equifinality phenomenon, which demonstrates the advantages of the MS-CUA method. Figure 3 showed the 95PPU of surface runoff in each iteration. The 95PPU bands were decreasing during each iteration. The uncertainty bands moved towards the observed runoff and tended to cover more observed results. SUFI-2 and GLUE were applied to the same case for comparison to evaluate the proposed method's performance.

(b) Comparison results of SUFI-2 and GLUE

### SUFI-2

The calibration and uncertainty analysis by using the SUFI-2 was firstly conducted. The NSE was used as the likelihood function, and the threshold value of the objective function was set to 0.40. The four iterations with 1000 simulations in each iteration were conducted. For a better comparison, the initial parameter ranges were set as the same as the parameter ranges for the first iteration of the MS-CUA method. The parameter range settings for each iteration were shown in Table 5. According to the evolution of parameter ranges in SUFI-2, the parameter ranges decreased during different iterations, and parameter ranges were centered on the best estimates^12^. The detailed statistics for each iteration were shown in Table 6. The R^2^ and NSE of the best-calibrated simulation were 0.78 and 0.75 in the fourth iteration, respectively indicating the good calibration results. The best-calibrated simulation results are slightly better than the fourth iteration calibrated results using the MS-CUA method (due to the higher NSE value). In the fourth iteration, the P/R ratio was 0.93, which was smaller than the P-factor and R-factor ratios of 1.00 in the fourth iteration of the MS-CUA method. Therefore, the SUFI-2 method can provide slightly better-calibrated simulation results, and the MS-CUA method can give more balanced uncertainty analysis results.

**Table 5:** Parameter range values of each iteration using SUFI-2.

Parameters	First iteration		Second iteration		Third iteration		Fourth iteration	
	LB	UB	LB	UB	LB	UB	LB	UB
CN_2_	− 0.25	0.30	− 0.40	0.10	− 0.40	− 0.02	− 0.30	− 0.10
ALPHA_BF	0.40	1.00	0.60	1.00	0.75	1.00	0.80	0.90
GW_DELAY	10	300	35	215	0	133	40	105
GWQMN	0	2000	0	1100	0	715	0	465
ESCO	0.80	1.00	0.87	1.00	0.91	1.00	0.94	1.00
CH_K	5	130	14	92	0	58	32	81
ALPHA_BNK	0.00	1.00	0.45	1.00	0.43	0.85	0.63	1.00
SOL_AWC	− 0.20	0.40	− 0.10	0.55	− 0.12	0.33	− 0.22	0.15
SFTMP	− 5.00	5.00	− 0.20	9.00	1.00	8.30	− 0.30	5.90
GW_REVAP	0.02	0.50	0.01	0.30	0.01	0.16	0.02	0.12
RCHRG_DP	0.00	1.00	0.00	0.50	0.00	0.30	0.00	0.16

**Table 6:** The statistic summary of the simulation results of each iteration using SUFI-2.

	Iteration	Threshold value	Behavioural parameter sets	P-factor	R-factor	R^2^	NSE	P/R
*SUFI-2*	First	0.40	31	0.31	0.99	0.74	0.63	0.31
	Second	0.40	214	0.36	0.89	0.80	0.72	0.40
	Third	0.40	741	0.56	0.92	0.79	0.76	0.61
	Fourth	0.40	1000	0.42	0.45	0.78	0.75	0.93
*GLUE*	One	0.60	28	0.39	0.51	0.79	0.70	0.76

To be noticed, if only three iterations have been conducted, the MS-CUA method can still provide reasonably good uncertainty analysis results (P/R = 0.93); at the same time, the SUFI-2 method only provided relatively poor uncertainty analysis results (P/R = 0.61). For example, Tables 3 and 6 showed that the results after the third iteration using MS-CUA already achieved similarly good results (P-factor value of 0.42, R-factor value of 0.45, R2 value of 0.75, NSE value of 0.73, and P/R = 0.93) with the results after the fourth iteration using SUFI-2 (P-factor value of 0.42, R-factor value of 0.45, R2 value of 0.78, NSE value of 0.75, and P/R = 0.93). Comparing with SUFI-2, MS-CUA is more efficient for searching the HPD regions due to the application of screening processes using additional criteria. When fewer computational resources were available, the MS-CUA method can make quick and reliable responses than the SUFI-2 method. Therefore, the MS-CUA method can more efficiently achieve a desired uncertainty analysis results with a reasonably well-calibrated simulation result.

### GLUE

GLUE was also used for calibration and uncertainty analysis for comparison purposes. A total of 10,000 simulation runs was conducted. The initial parameter ranges for the MS-CUA method were used as the parameter ranges for each parameter in GLUE. The NSE was used as the likelihood function to keep consistency with the other two methods, and the threshold value was set to 0.60 because a large number of simulation runs was conducted. Table 6 shows the calibration and uncertainty analysis results by using the GLUE method. As shown in Table 6, there were only 28 behavioural parameter sets among the total of 10,000 parameter sets, representing the low efficiency of searching the optimal results using GLUE. The R^2^ and NSE value were 0.79 and 0.70, respectively, which were smaller than the results from the fourth iteration of the MS-CUA method (R^2^ = 0.79 and NSE = 0.74). The P/R ratio was 0.76 lower than the value of the MS-CUA method (P/R = 1.00), indicating that the proposed method can provide a better-calibrated simulation with better uncertainty analysis results. Meanwhile, totally 4000 simulation runs were conducted using MS-CUA to achieve the better calibration and uncertainty analysis results in comparison with the 10,000 simulation runs by GLUE. Consequently, MS-CUA had significantly reduced time (by 60%) with the better solutions showing its strength and efficiency.

According to the results from the three methods, it was clearly shown that the MS-CUA method could reach similar good calibration results with SUFI-2 (which was better than GLUE) and provided the best uncertainty analysis results among the three methods. The MS-CUA method also showed the ability to efficiently search the HPD region and reduce the prediction uncertainty of surface runoff and parameter uncertainty while demonstrating its advantages over other methods.

## Conclusions

In this study, a developed calibration and uncertainty analysis method (MS-CUA) was proposed for hydrological modeling studies. The MS-CUA method with the proposed framework aimed to calibrate hydrological models and provide balanced and reliable uncertainty analysis results in a high-efficient way. The feasibility and flexibility of MS-CUA method were evaluated through two case studies based on the simulations from the SWAT model. A comprehensive analysis was achieved according to the hypothetical case results with demo data from SWAT-CUP and the real case study at upstream of the Wenjing River watershed.

In the hypothetical case study, the results indicated that with 2000 simulation runs, the MS-CUA method can achieve better values of R^2^, NSE and P/R ratio than those in 10,000 simulation runs by GLUE. In the real-world case study, the 4000 simulation runs using MS-CUA were compared with 4000 simulation runs using SUFI-2 and 10,000 simulation runs using GLUE. The MS-CUA method can more efficiently provide better uncertainty analysis results than the SUFI-2 method with similarly good calibration results. The MS-CUA method obtained better calibration results and much better uncertainty analysis results with fewer simulation runs with the comparison of GLUE. The results indicated that the MS-CUA method can search optimal simulations and provide balanced uncertainty analysis results with high efficiency.

Through the case studies, the results showed that the MS-CUA method was able to locate the HPD regions of each parameter fast for the improvement of the computational efficiency and reduction of the parameter uncertainty without sacrificing the simulation performance for surface runoff prediction. The MS-CUA method could be applied to the high-dimensional parameter estimation problems and complex simulation models due to the high computational efficiency. In this study, using the LHS method is an example of using advanced sampling methods to improve the efficiency of calibration and uncertainty analysis. As one of the advantages of the proposed MS-CUA method which can integrate/adopt up-to-date and new efficient sampling methods to improve the performance, more sampling schemes could be adopted using MS-CUA in future studies.

Through the addition criterion (R^2^) application, behavioural parameter sets were further screened for the refined behavioural parameter sets in each iteration. The reduced number of behavioural parameter sets can decrease the influence of equifinality phenomenon showing the unique advantage of the MS-CUA method. The parameter ranges in the MS-CUA method are always centered on the best simulation results and narrowed down from the original ranges with the use of refined behavioural parameter sets. Thus, the parameter uncertainty has been reduced after each iteration. The reduced parameter uncertainty was quite important when conducting the uncertainty analysis for propagation effects. The less uncertainty from the source would lead to much smaller total uncertainty after propagation for future studies. The proposed MS-CUA approach also has the potential to deal with the problems related to calibration and uncertainty analysis of other numerical simulation models.

## Methods

- **The MS-CUA method**

**Figure 4:**
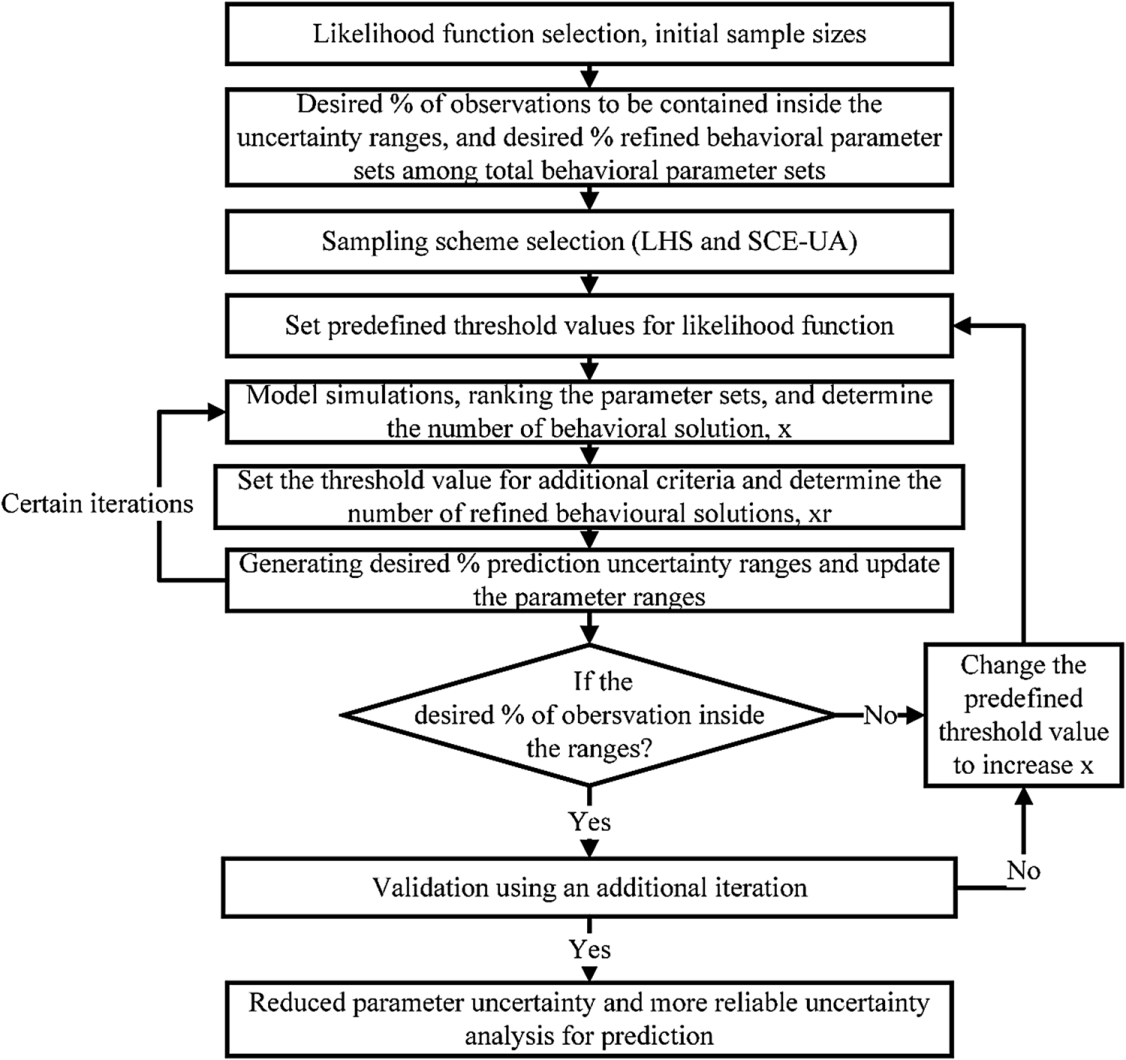
The framework of the MS-CUA method for calibration and uncertainty.

Using advanced sampling methods for prior distributions and new criteria for screening behavioural parameter sets for posterior distributions, narrower and more reliable parameter uncertainty ranges could be provided for improving the simulation performance. The framework (Fig. 4) and detailed procedures of the MS-CUA method are shown as follows:

**Step 1**: ***The selection of likelihood functions***. As a popular likelihood function, Nash–Sutcliffe Efficiency (NSE) (Eq. 1) can be selected^38,39^. The initial sample sizes should be defined at this step. Due to the application of multiple iterations and high-efficiency sampling methods, relatively small sample sizes for each iteration are used.

1 $$NSE = {1 - }\frac{{\sum\limits_{i = 1}^{n} {\left( {Q_{s,i} - Q_{o,i} } \right)^{2} } }}{{\sum\limits_{i = 1}^{n} {\left( {Q_{o,i} - \overline{{Q_{o} }} } \right)^{2} } }}$$

where *Q*_*o,i*_ and *Q*_*s,i*_ are the observed and simulated values on day *i*, respectively; ${\overline{Q}}_{o}$ and ${\overline{Q}}_{i}$ are the average values of the observed and simulated surface runoff (m^3^/s), respectively; and *n* is the total number of values within the period of analysis.

**Step 2**: ***The definition of acceptable percentage levels.*** Decide the percentage of observations within uncertainty ranges and the percentage of refined behavioural parameter sets among total behavioural parameter sets. If the selected number of behavioural parameter sets is too small, the parameter ranges for the next iteration may not be accurate. Therefore, an appropriate percentage of behavioural parameters is necessary to ensure a reasonably good estimation for the next iteration.

**Step 3**: ***The determination of  parameter ranges.*** Parameter ranges can be assigned according to the physical meaning and current understanding of parameters. And then, advanced sampling schemes methods can be applied. Due to missing information, the prior distributions of parameters are generally assumed as uniform distributions. It is a typical assumption in hydrological modeling, because the prior distribution form of parameters was normally difficult to determine^40^.

**Step 4**: ***The definition of threshold values of the likelihood function.***. The threshold values of the likelihood function are defined for screening the behavioural and non-behavioural parameter sets. A higher value of the objective function threshold typically achieves smaller numbers of behavioural parameter sets. For different iterations, multiple choices of likelihood function values can be applied for a better estimation of parameter ranges. For example, relatively low threshold values can be selected for the first iteration due to the lack of information.

**Step 5: *The computation of hydrological simulation and likelihood function values***. Hydrological simulations can be conducted using predefined parameter sets. And then, the likelihood function values of simulations from each parameter set are calculated. The simulation results from parameter sets are further ranked based on their likelihood values. The parameter sets with likelihood function values lower than the threshold value are removed. The remaining parameter sets are defined as behavioural parameter sets.

**Step 6**: ***The application of additional criteria.*** Additional criteria are to screen out the "bad" behavioural simulations, even though their NSE values are greater than the predefined threshold value. After removing the behavioural parameter sets with the additional criteria, the remaining parameter sets are called "refined behavioural parameter sets". For example, the coefficient of determination (*R*^2^) can be used as an additional criterion for screening refined behavioural parameter sets (Eq. 2). Moreover, with the involvement of new constraints with new information (e.g., new observations can lead to changes of some parameter ranges), some behavioural parameter sets will be removed. Eventually, the number of refined behavioural parameter sets will be reduced and determined, and prediction uncertainty will be reduced and quantified correspondingly. By using the additional criteria, the influence of equifinality phenomenon can be controlled and reduced through the refining process using the above steps. In certain cases, some attempts of additional criteria (Eqs. 2, 3, and 4) can be considered when data are available as follows:

2 $$R^{2} = \frac{{\left[ {\sum\limits_{i = 1}^{n} {(Q_{o,i} - \overline{{Q_{o} }} )\left( {Q_{s,i} - \overline{{Q_{s} }} } \right)} } \right]^{2} }}{{\sum\limits_{i = 1}^{n} {(Q_{o,i} - \overline{Q}_{o} )^{2} \sum\limits_{i = 1}^{n} {(Q_{s,i} - \overline{Q}_{s} )^{2} } } }}$$

3 $$L_{1} = \frac{1}{{n_{p} }}\sum\limits_{j = 1}^{{n_{p} }} {\left| {\frac{{Q_{o,j} - Q_{s,j} }}{{Q_{o,j} }}} \right|}$$

where *n*_*p*_ is the number of peak flows during the study period. It can be assumed that the average difference between observed peak flow and simulated peak flow cannot be more than 30% for a behavioural simulation. Therefore, for parameter sets that have *L*_1_ the greater than 30%, these parameter sets will be removed from total behavioural parameter sets. The remaining parameter sets are considered refined behavioural parameter sets.

4 $$L_{2} = \frac{1}{{n_{p} }}\sum\limits_{j = 1}^{{n_{p} }} {\left| {t_{o,j} - t_{s,j} } \right|}$$

where *t*_*o,j*_ is the observed time for accessing the peak flow (hour), *t*_*s,j*_ is the simulated time for accessing peak flow (hour). For example, if the difference between the time to peak flow in observation and in simulation results is assumed than less than 3 h. In that case, any parameter sets making *L*_*2*_ greater than 3 h will be removed to obtain the refined behavioural parameter sets.

**Step 7**: ***The analysis of model prediction uncertainty.*** Model prediction uncertainty analysis can be conducted in this step. The lower and upper bounds of model prediction results are determined for surface runoff. By sorting the likelihood values of simulations from each parameter sets, the time series surface runoff prediction uncertainty under the given confidence level could be estimated. For most studies, 2.5% and 97.5% of the cumulative distribution of surface runoff are set as the lower and upper bounds as prediction uncertainty, respectively. Therefore, 95PPU of the surface runoff simulation is obtained through the analysis. For the next iteration, the parameter ranges are updated accordingly using the parameter ranges for the simulations achieving the 95PPU of surface runoff. Since the number of refined behavioural parameter sets is continuously reduced after each iteration, the corresponding parameter ranges will be further reduced. After the update of parameter ranges, go back to **Step 5** for the new iteration till the desired numbers of iteration are completed.

**Step 8: *The determination of the desired percentage of observation.*** When 95PPU values of surface runoff are obtained, the desired percentage of observation within the 95PPU can be determined. If the percentage is too low and cannot meet the pre-set value, go back to **Step 4**: change the threshold value of the likelihood function to a lower one or adjust the critical value of other criteria to increase the behavioural parameter sets for getting a reasonable estimation of parameter ranges. If the percentage of observation becomes reasonable, the next step can proceed.

**Step 9**: ***The validation of the proposed method.*** Validation of the proposed method is necessary. After obtaining the parameter ranges from the last iteration of simulations, the newly updated parameter ranges are applied for one more iteration to check if the reasonable outcomes are achieved. If simulation results are acceptable, go to the last step; if not, go back to **Step 4**.

**Step 10: *The results of calibration and uncertainty analysis***. This proposed method uses multiple iterations with advanced sampling methods and screens out the "bad" behavioural parameter sets using additional criteria to efficiently and accurately search the optimal results with consideration of uncertainty. The reduced parameter ranges also lead to smaller parameter uncertainty reflected by 95PPU of the surface runoff. The highly efficient calibration and uncertainty analysis can be achieved.

The MS-CUA method is different from the GLUE method, which accepts all the parameter sets as behavioural parameter sets if the likelihood values are greater than the predefined threshold value. To our knowledge, limited studies have focused on refining the posterior distribution of parameters to make the simulation more accurate. The GLUE method generally applies only one likelihood function, which should increase monotonically with the similarity in behavior increase (e.g., Nash–Sutcliffe coefficient)^12^. The objective of the GLUE method is to identify a set of behavioural models within the range of possible parameter combinations. The term "behavioural" indicates acceptable models based on available data and knowledge. Therefore, a threshold value needs to be predefined before screening the behavioural and non-behavioural parameter sets. However, some parameter sets can achieve a high value for the likelihood function but are still not reasonable in practice. Because the NSE only evaluates the goodness-of-fit of the overall runoff simulation, some impractical and inaccurate simulation results are involved in behavioural results. For example, it could have an NSE value over 0.80 for the overall simulation for runoff, but the simulated peak flow is quite different from the observed peak flow; or the time for the peak flow in simulation is much different from the time for the corresponding peak flow in observation. On the other hand, similar to SUFI-2, MS-CUA implement multiple iterations for the performance improvements of simulation and uncertainty analysis. However, different updating algorithms of parameter ranges are applied in each iteration.

- **Two sampling schemes**

In the proposed methods, instead of using the Monte Carlo random sampling method for prior distribution sampling of model parameters, advanced sampling methods are applied for parameter prior distributions to improve sampling efficiency. The sampling methods can be LHS and shuffled complex evolution (SCE-UA).

- Latin hypercube sampling (LHS)

The LHS method is a probabilistic procedure with the combinations of multiple desired features of random sampling and stratified sampling for more stable analysis outcomes rather than random sampling^41^. Many studies have proved that the LHS method is an efficient way for assessing output uncertainty of models (e.g., most distributed hydrological models) with multiple parameters^42–44^. This technique applies a stratified sampling scheme, which allows an efficient description of the output. The standard LHS method contains three major steps as follow^45^:

- **Step 1**: ***Equiprobable subdivision***: The probability distribution of parameters in each model is subdivided into *T* ranges/intervals with a probability of occurrence equal to *1/T,* respectively.
- **Step 2**: ***Stratified sampling***: A single value is sampled within each range/interval according to the probability distribution (normally, the uniform distribution is assumed for parameters).
- **Step 3**: ***Random pairing***: *T* data sets of *p* parameters (*p* is the number of parameters to be sampled) are created.

The LHS method represents a better performance with a more uniform coverage of parameter space than other methods in estimating the statistics of a population of function with less model simulations^46^. Moreover, the LHS method is easy to be implemented. Therefore, using the LHS method, the high-efficient sampling for uncertainty analysis can be relatively easy to achieve.

(b) Shuffled complex evolution (SCE-UA)

The SCE-UA algorithm, developed by Duan et al.^47^, is a robust global search algorithm to sample parameter sets for prior distribution. Originally, this method is used to minimize a single objective function on the basis of four concepts: (1) the combination of deterministic and probabilistic methods; (2) the ability for a systematic evolution for the parameter space to the global optimum; (3) multiple competitive evolutions; (4) complex shuffling in each community. Because of these features, the SCE-UA method is effective, robust, and flexible. The major steps of the SCE-UA method are described below^48,49^:

- **Step 1**: ***Generate samples:***: Sample *s* points randomly in the reasonable parameter range and compute the likelihood value at each point. Typically, the uniform probability distribution will be applied for the sample generation if lacking prior information.
- **Step 2**: ***Rank samples***: Sort the *s* points in ascending order of likelihood values.
- **Step 3**: ***Partition into complexes***: Partition *s* points into *p* groups (called complexes), and each complex contains *m* points. The complexes are partitioned so that the first complex contains every *p(k-1)*+*1* ranked point, the second complex contains every *p(k-1)*+*2* ranked point and so on, where *k*=*1,2,…,m.*
- **Step 4**: ***Evolve each complex***: Evolve each complex according to the competitive complex evolution (CCE) algorithm.
- **Step 5**: ***Shuffle complexes***: combine the points in the evolved complexes into a single sample population and sort the sample population by ascending sequence according to likelihood values.
- **Step 6**: ***Check convergence***: If any of the pre-specified convergence criteria are satisfied, then stop; otherwise, then continue.
- **Step 7**: ***Check the reduction of the number of complexes***: If the minimum number of complexes required in a population (*p*_*min*_) is less than *p,* remove the lowest-ranked complex; set *p*=*p-1* and *s*=*pm*; return to Step 4; If *p*_*min*_=*p,* go back to Step 4.

By using the SCE-UA method, considerable improvements can be made due to an adaptive sampling method that uses information from past draws to update the search direction. In such a way, SCE-UA can generate a more robust and efficient estimation for parameters and prediction uncertainty in comparison to a traditional random Monte Carlo sampling method.

- Hydrological modeling

The hydrological modeling was conducted using the soil and water assessment tool (SWAT), and the simulation results were used for analysis in this study. SWAT is a continuous-time, spatially distributed hydrological model. It was first developed by the United States Department of Agriculture–Agricultural Research Service (USDA-ARS)^50^. SWAT can help water resource managers predict the impacts of land management practice on water, sediment, and agricultural chemical yields^51,52^. The SWAT model uses watershed information (e.g. weather, soil, topography, vegetation and land management practices) to simulate watershed hydrological processes such as surface and subsurface runoff, water quality, erosion and sedimentation^53^. One of the advantages of the SWAT model is the capability of simulations in ungaged river basins. By considering data availability, SWAT has been selected for this case study.
